# PIF transcription factors-versatile plant epigenome landscapers

**DOI:** 10.3389/freae.2024.1404958

**Published:** 2024-05-16

**Authors:** Moonia Ammari, Kashif Maseh, Mark Zander

**Affiliations:** Department of Plant Biology, Waksman Institute of Microbiology, Rutgers, The State University of New Jersey, Piscataway, NJ, United States

**Keywords:** plant epigenomics, chromatin dynamics, transcription factors, chromatin remodeling complexes, light and temperature signaling

## Abstract

Plants are exquisitely responsive to their local light and temperature environment utilizing these environmental cues to modulate their developmental pathways and adjust growth patterns. This responsiveness is primarily achieved by the intricate interplay between the photoreceptor phyB (phytochrome B) and PIF (PHYTOCHROME INTERACTING FACTORs) transcription factors (TFs), forming a pivotal signaling nexus. phyB and PIFs co-associate in photobodies (PBs) and depending on environmental conditions, PIFs can dissociate from PBs to orchestrate gene expression. Until recently, the mechanisms governing epigenome modifications subsequent to PIF binding to target genes remained elusive. This mini review sheds light on the emerging role of PIFs in mediating epigenome reprogramming by recruiting chromatin regulators (CRs). The formation of numerous different PIF-CR complexes enables precise temporal and spatial control over the gene regulatory networks (GRNs) governing plant-environment interactions. We refer to PIFs as epigenome landscapers, as while they do not directly reprogram the epigenome, they act as critical sequence-specific recruitment platforms for CRs. Intriguingly, in the absence of PIFs, the efficacy of epigenome reprogramming is largely compromised in light and temperature-controlled processes. We have thoroughly examined the composition and function of known PIF-CR complexes and will explore also unanswered questions regarding the precise of locations PIF-mediated epigenome reprogramming within genes, nuclei, and plants.

## Introduction

The major sensor of red (R), far-red (FR) light as well temperature in *Arabidopsis* is the photoreceptor phyB (Quail et al., 1995; Rockwell et al., 2006; Jung et al., 2016; Legris et al., 2016). After photoactivation, phyB translocates from the cytosol to the nucleus where it compartmentalizes into PBs through liquid-liquid phase separation (LLPS) (Sakamoto and Nagatani, 1996; Kircher et al., 1999; Chen et al., 2003; Chen et al., 2022). PBs function as regulatory light and temperature modules, housing a variety of signaling components that interact directly or indirectly with phyB (Van Buskirk et al., 2012; Jung et al., 2016; Legris et al., 2016; Hahm et al., 2020; Pardi and Nusinow, 2021; Kim et al., 2023; Kwon et al., 2024; Willige et al., 2024). One of the most critical PB component are the PIFs, a family of basic helix-loop-helix (bHLH) TFs which acts a transcriptional activators or repressors (Ni et al., 1998; Huq and Quail, 2002; Huq et al., 2004; Oh et al., 2004; Castillon et al., 2007; Leivar et al., 2008; Shen et al., 2008; Leivar and Quail, 2011; Leivar and Monte, 2014). The *Arabidopsis* genome encodes eight *PIFs* (*PIF1-PIF8*), which integrate phyB’s environmental input into GRNs underlying light and temperature responses (Leivar and Quail, 2011; Jeong and Choi, 2013; Leivar and Monte, 2014; Pham et al., 2018; Bian et al., 2022; Han et al., 2023). PIF1/3/4/5 control the transition from skotomorphogenesis (SM) to photomorphogenesis (PM) by repressing light-responsive gene expression in the dark until light exposure initiates their phytochrome-mediated phosphorylation, ubiquitination and subsequent proteasomal degradation (Leivar and Quail, 2011; Leivar and Monte, 2014; Pham et al., 2018). Furthermore, PIF7 functions as the major regulator alongside PIF1/3/4/5 in orchestrating the shade avoidance syndrome (SAS) (Willige et al., 2021). This syndrome is induced by low R:FR light ratios (referred to as shade) resulting from nearby vegetation, prompting elongation of hypocotyls and petioles, early flowering, and upward leaf positioning (Franklin and Whitelam, 2005; Franklin, 2008; Casal, 2012; Sessa et al., 2018; Casal and Fankhauser, 2023). A phenotypically similar response to SAS is thermomorphogenesis (TM) which refers to the profound effect of elevated ambient temperature (EAT) (up to 28°C, below the heat stress range) on plant growth, development, and immunity (Casal and Balasubramanian, 2019; Burko et al., 2022). In *Arabidopsis*, TM regulation primarily involves PIF4 and PIF7 (Koini et al., 2009; Kumar et al., 2012; Gangappa et al., 2017; Chung et al., 2020; Fiorucci et al., 2020) whereas TM under shade (low R:FR light) conditions is mainly regulated by PIF7 alone (Burko et al., 2022).

The detailed complex mechanisms governing PIFs at both the transcriptional and protein levels along the crosstalk with other signaling pathways have been extensively discussed in several excellent reviews (Leivar and Quail, 2011; Jeong and Choi, 2013; Shin et al., 2013; Leivar and Monte, 2014; Paik et al., 2017; Pham et al., 2018; Favero, 2020). Additionally, readers are encouraged to explore superb reviews that delve into the exciting connection between light/temperature signaling and chromatin dynamics (Perrella and Kaiserli, 2016; Bourbousse et al., 2019; Jing and Lin, 2020; Perrella et al., 2020). We aim to provide a brief introduction to PIF7, which serves as a partial representation of other PIFs and highlights crucial regulatory aspects such as posttranslational modifications (PTMs), condensate formation, and DNA binding among PIF proteins. In white light (WL) PIF7 is phosphorylated and unlike other PIFs relatively light-stable (Leivar et al., 2008; Huang et al., 2018; Willige et al., 2021; Zhou et al., 2021). PIF7 undergoes LLPS to form biocondensates under WL conditions which subsequently associate with phyB condensates in photobodies (PBs) (Leivar et al., 2008; Willige et al., 2021; Chen et al., 2022; Xie et al., 2023). Upon exposure to shade, PIF7 gets rapidly dephosphorylated and dissociates from PBs to bind to G-boxes (CACGTG) within *cis*-regulatory elements (CREs) of its targets (Chung et al., 2020; Willige et al., 2021; Xie et al., 2023). PIF7 regulates an extensive GRN encompassing multiple biosynthesis genes for the growth-promoting plant hormone auxin, along with numerous transcription factors (TFs) such as *ATHB2* (*ARABIDOPSIS THALIANA HOMEOBOX PROTEIN 2)* (Chung et al., 2020; Willige et al., 2021). A similar signaling mechanism operates during TM (Chung et al., 2020; Fiorucci et al., 2020; Burko et al., 2022), where PIF7 mRNA also serves as a direct thermosensor (Chung et al., 2020).

Here, we will discuss the emerging role of PIFs as epigenome landscapers by recruiting various chromatin regulators (CRs) to shape the epigenome at their target genes (Figure 1). The plant epigenome plays a crucial role as a regulatory framework, integrating both developmental signals and environmental cues into spatiotemporal-specific GRNs (Lloyd and Lister, 2022). In a broad sense, the epigenome encompasses not only all chemical modifications of DNA and histone proteins but also other features that control gene expression, including DNA binding of TFs and CRs, chromatin accessibility, 3D chromatin conformation, nucleosome positioning, and long non-coding RNAs (Rivera and Ren, 2013). We utilize the term CR to encompass all regulatory factors capable of modifying the epigenome, chromatin remodeling complexes (CRCs), histone acetyltransferases/deacetylases (HATs/HDACs), histone methyltransferases/demethylases (KMTs/KDMs), and many others. To enhance comprehension, we discuss various epigenome feature dynamics separately, although it is crucial to recognize their intricate interconnections. Finally, we outline some unresolved questions for further exploration.

**FIGURE 1:**
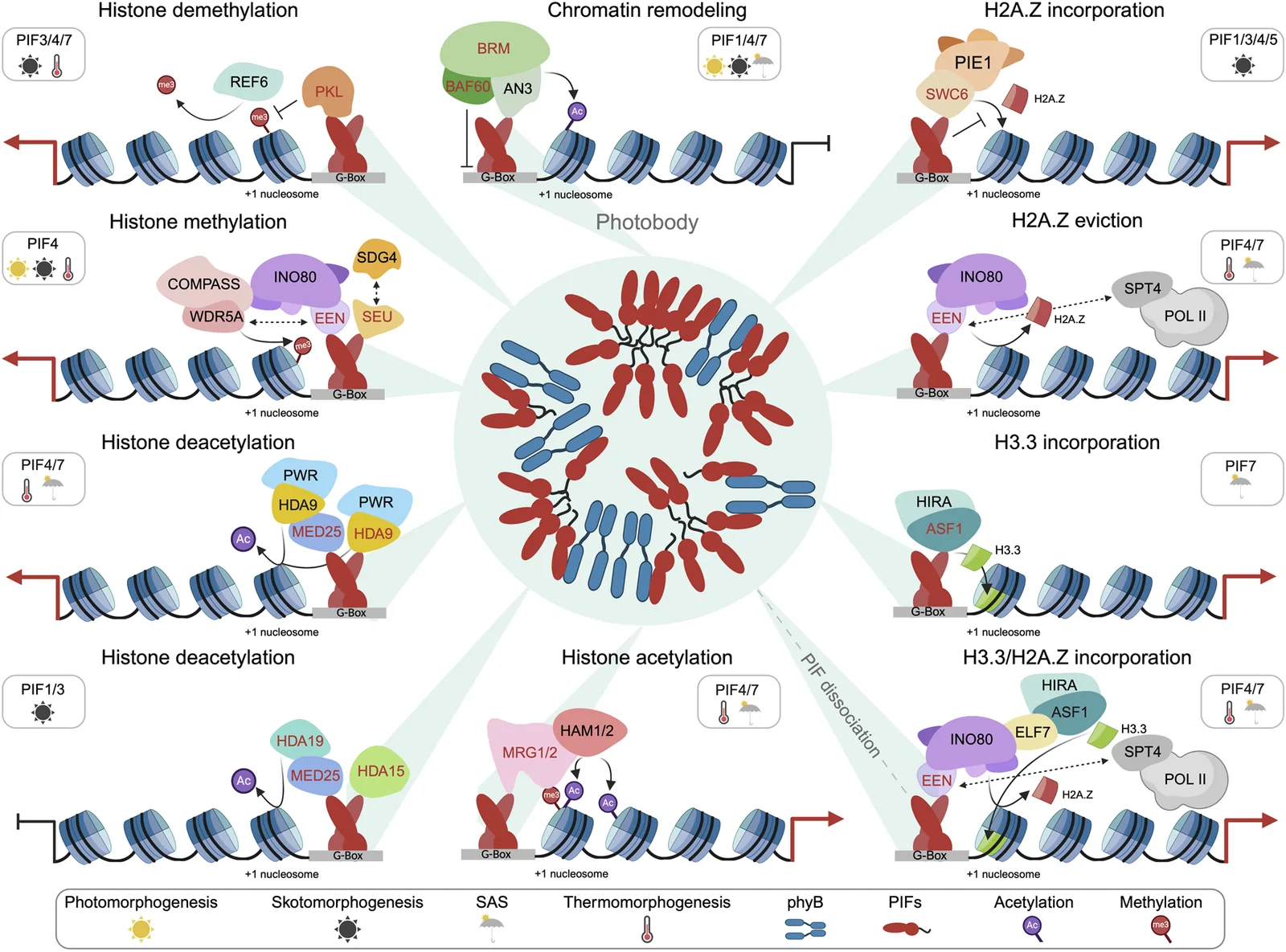
The illustration provides an overview of the current understanding of different PIF-CR complexes. The PBs containing phyB-PIF act as a reservoir for PIFs, enabling their dissociation based on environmental cues. Various PIF-controlled features are depicted. While the complexes are presented separately for clarity, they likely function concurrently. The respective PIFs implicated in specific epigenomic features are highlighted, along with symbols denoting the four major processes (SM, PM, SAS, and TM) where the PIF-CR complexes act. EEN-SPT4 and EEN-WDR5A interactions are shown as dashed double arrows for better visualization. Abbreviations: AN3 (ANGUSTIFOLIA3), ASF1 (ANTI-SILENCING FUNCTION 1A/B), BAF60 (BRG1/BRM associated factor 60), BRM (BRAHMA), COMPASS (Complex Proteins Associated with Set1), EEN (EIN6 ENHANCER), ELF7 (EARLY FLOWERING 7), HAM1/2 (HISTONE ACETYLTRANSFERASE OF THE MYST FAMILY 1 and 2), HDA9 (HISTONE DEACETYLASE 9), HDA15 (HISTONE DEACETYLASE 15), HDA19 (HISTONE DEACETYLASE 19), HDA9 (HISTONE DEACETYLASE 9), HIRA (HISTONE REGULATORY HOMOLOG A), INO80 (INOSITOL REQUIRING 80), MED25 (MEDIATOR 25), MRG1/2 (MORF-RELATED GENE 1/2), PIE1 (PHOTOPERIOD-INDEPENDENT EARLY FLOWERING 1), PKL (PICKLE), POL II (RNA Polymerase II), PWR (POWERDRESS), REF6 (RELATIVE OF EARLY FLOWERING 6), SDG4 (SET DOMAIN GROUP 4), SEU (SEUSS), SPT4 (Suppressor of Ty 4), SWC6 (SWR1 complex subunit 6), and WDR5A (WD40 REPEAT 5).

### H2A.Z and H3.3 dynamics

Histone variants are histone proteins that differ from canonical histones in their amino acid sequence, structure, and functions (Yi et al., 2006; Borg et al., 2021). One of the best studied histone variants in *Arabidopsis* is H2A.Z that confers gene responsiveness to environmentally-responsive genes (Coleman-Derr and Zilberman, 2012). The *Arabidopsis* genome encodes three functionally redundant H2A.Z genes (*HTA8*, *HTA9*, *HTA11*) whose mutations leads to pleiotropic effects such as early flowering, enhanced disease resistance and DNA hypermethylation (Choi et al., 2007; March-Diaz et al., 2008; Coleman-Derr and Zilberman, 2012; Nie et al., 2019). H2A.Z exerts a repressive influence on transcription, and its eviction is a common characteristic of transcriptional activation in plant-environment interactions (Kumar and Wigge, 2010; Kumar et al., 2012; Boden et al., 2013; Cortijo et al., 2017; Sura et al., 2017; Zander et al., 2019; Willige et al., 2021; Xue et al., 2021).

The discovery of H2A.Z’s involvement in PIF-regulated processes occurred with a forward genetic screen aiming to identify temperature sensors in *Arabidopsis* (Kumar and Wigge, 2010). This screen revealed ARP6 (ACTIN-RELATED PROTEIN 6), a subunit of the SWR1 (SWI2/SNF2 (SWITCH/SUCROSE NONFERMENTABLE)-related 1) chromatin remodeler (Choi et al., 2005; Martin-Trillo et al., 2006; Choi et al., 2007), as a negative TM regulator (Kumar and Wigge, 2010). Other key components of the multi-subunit plant SWR1 complex (SWR1-C) are the ATPase PIE1 (PHOTOPERIOD-INDEPENDENT EARLY FLOWERING 1) and accessory units such as SWC6 (SWR1 COMPLEX 6) and SEF (SERRATED LEAVES AND EARLY FLOWERING) (Noh and Amasino, 2003; Choi et al., 2007; March-Di et al., 2007; Luo et al., 2020). Consistent with SWR1-C’s role in incorporating H2A.Z into chromatin (Deal et al., 2007), *arp6* mutants have reduced H2A.Z levels resulting in the elevation of thermo-responsive gene expression and longer hypocotyls and petioles even without an EAT stimulus (Kumar and Wigge, 2010). As PIF4 governs the expression of EAT-induced genes (Koini et al., 2009), it was suggested that H2A.Z-containing nucleosomes occlude PIF4 from binding to its target genes. This hindrance is lifted in *arp6* mutants, thereby facilitating higher thermo-responsive gene expression (Kumar and Wigge, 2010).

The first direct connection between H2A.Z and PIFs was shown for PIF4-dependent EAT-induced flowering in *Arabidopsis* (Kumar et al., 2012). EAT-induced expression of *FT* (*FLOWERING LOCUS T*) requires direct PIF4 binding at *FT* which is facilitated by EAT-induced eviction of H2A.Z nucleosomes (Kumar et al., 2012). To elucidate the PIF-H2A.Z interplay in more detail, PIF7 DNA binding as well as H2A.Z occupancy in wildtype and *pif457* mutants were tracked simultaneously during SAS over time using ChIP-seq (Chromatin immunoprecipitation followed by sequencing) (Willige et al., 2021). PIF7 initiates shade-induced H2A.Z eviction at its target genes through the association with the INO80 (INOSITOL REQUIRING 80) chromatin remodeling complex (INO80-C) via direct interaction with its essential subunit EEN (EIN6 ENHANCER) (Zander et al., 2019; Willige et al., 2021). Strikingly, this PIF-INO80-C regulatory module is also operational with PIF4 during TM (Figure 1) (Sureshkumar and Balasubramanian, 2021; Xue et al., 2021).

INO80-C belongs to the INO80-type subfamily of SWI2/SNF2 chromatin remodeler (Clapier and Cairns, 2009; Han et al., 2015) and it was shown in various species that INO80-C can facilitate H2A.Z eviction to regulate gene expression (Papamichos-Chronakis et al., 2011; Alatwi and Downs, 2015; Brahma et al., 2017; Zhao et al., 2022). In plants, INO80-C’s role in H2A.Z eviction was shown for ethylene-, low R:FR light-, and EAT-induced genes (Zander et al., 2019; Willige et al., 2021; Xue et al., 2021). INO80-C additionally acts as a platform for recruiting other CRs, thereby potentially expanding the functional capabilities of the PIF-INO80-C regulatory module (Shang et al., 2021; Xue et al., 2021; Zhao et al., 2023). Its subunit EEN interacts with WDR5a (WD40 REPEAT 5), an integral component of the COMPASS (Complex Proteins Associated with Set1) histone H3K4 methyltransferase complex, to facilitate trimethylation of histone 3 lysine 4 (H3K4me3) at PIF4 target genes (Figure 1) (Xue et al., 2021). The association of INO80-C with other major COMPASS histone H3K4 methyltransferase complex components was independently shown (Shang et al., 2021). In addition, EEN interacts with the transcription elongation factors (TEFs) SPT4 (Suppressor of Ty 4)-1 and SPT4-2 to mediate RNA Polymerase II (RNAPII) elongation during TM (Figure 1) (Xue et al., 2021). Interestingly, PIF1/3/4/5 can also associate with SWR1-C via SWC6 during PM to inhibit H2A.Z deposition (Figure 1) (Chen H. et al., 2023). Under this scenario, PIFs inhibit SWR1-C activity at auxin-responsive genes in the dark through an unknown mechanism (Chen H. et al., 2023). All these findings indicate that PIFs can use multiple strategies to alter the H2A.Z landscape at their target genes thereby fine-tuning their expression in an environmental stimulus-dependent manner.

The histone variant H3.3 forms together with the replicative H3.1/H3.2, and the centromeric CenH3 the H3 (Histone 3) family (Henikoff and Ahmad, 2005; Borg et al., 2021). H3.3 is incorporated during transcription and rapid upregulation of environmentally-responsive genes is compromised in *h3.3* knockdown mutants (Wollmann et al., 2017). The deposition of H3.3 is in part mediated by the histone chaperones ASF1A (ANTI-SILENCING FUNCTION 1A) and ASF1B in conjunction with the histone chaperone HIRA (HISTONE REGULATORY HOMOLOG A) (Tagami et al., 2004; Zhu et al., 2011; Nie et al., 2014; Duc et al., 2015; Zhong et al., 2022). During SAS, PIF7 recruits ASF1A/B and HIRA to facilitate H3.3 deposition at shade-responsive genes (Figure 1) (Yang et al., 2023). In addition, *asf1ab* and *hira* mutants show also reduced hypocotyl elongation under EAT (Yang et al., 2023; Zhao et al., 2023) which suggest that the PIF-ASF1A/B-HIRA module is also operational during TM. Although a PIF4-ASF1A/B interaction has not been confirmed, ASF1A/B could also be indirectly recruited by PIF4 through the INO80 ATPase that associates with ASF1A/B through the TEF PAF1c (Polymerase-Associated Factor 1 complex) subunit ELF7 (EARLY FLOWERING 7) (Figure 1) (Zhao et al., 2023). These findings highlight again the prominent role of PIFs in initiating epigenomic reprogramming through the recruitment of functionally diverse CRs.

### Histone acetylation dynamics

One of the most extensively studied PTM of histones is acetylation, which plays a crucial role in numerous gene regulatory processes (Jiang et al., 2020; Shvedunova and Akhtar, 2022; Chen Y. et al., 2023). Acetylation occurring at various lysine residues of histones H3 and H4, such as H3K9ac or H3K27ac, typically corresponds to gene activation, whereas deacetylation is associated with gene repression (Pandey et al., 2002). The balance of histone acetylation is regulated by the interplay between HATs and HDACs (Eberharter and Becker, 2002). The *Arabidopsis* genome encodes for 12 HATs and 18 HDACs (Pandey et al., 2002; Hollender and Liu, 2008), and although no direct interactions between PIFs and HATs have been documented, an active role of HATs in PIF-mediated chromatin reprogramming can be inferred (Martínez-García and Moreno-Romero, 2020). During SAS, H3K9ac levels rapidly increase at gene bodies of shade-responsive genes in a PIF4/5/7-dependent manner (Willige et al., 2021). Furthermore, levels of H4K5ac, H3K9ac, and H3K27ac increase in response to shade and EAT at *YUC8* in a PIF4/7-dependent manner (Peng et al., 2018; Zhou et al., 2024).

PIF4/7 directly associate with MRG1/2 (MORF-RELATED GENE 1/2) which are histone methylation readers that bind to H3K4/H3K36 trimethylation (H3K4me3/H3K36me3) and can interact with the HATs HAM1/2 (HISTONE ACETYLTRANSFERASE OF THE MYST FAMILY 1 and 2) (Xu et al., 2014). The recruitment of HAM1/2 through the PIF4/7-MRG1/2 module is the current model of PIF4/7-driven histone acetylation dynamics during SAS and TM (Figure 1) (Peng et al., 2018; Zhou et al., 2024). However, additional experimental support is needed because no direct association of HAM1/2 with PIF target genes has been shown so far (Latrasse et al., 2008).

HDA9 (HISTONE DEACETYLASE 9) was found to positively regulate hypocotyl elongation during TM and SAS (Tasset et al., 2018; van der Woude et al., 2019; Nguyen et al., 2023). HDA9’s positive role is still puzzling since expression of *PIF4* and *YUC8*, both essential regulators of EAT-induced hypocotyl elongation (Koini et al., 2009; Franklin et al., 2011; Sun et al., 2012; Proveniers and van Zanten, 2013; Bellstaedt et al., 2019), requires HDA9-mediated H3K9ac/14ac deacetylation at its +1 nucleosome (van der Woude et al., 2019). Interestingly, mutation of the HDA9-interacting SANT-domain containing protein PWR (POWERDRESS), phenocopies *hda9* mutants regarding reduced *PIF4/YUC8* expression due to higher H3K9ac levels (Tasset et al., 2018). EAT-induced H2A.Z eviction was also compromised in *hda9* mutants (van der Woude et al., 2019), however, the mechanism of how HDA9 regulates H2A.Z eviction is still unclear. Although PIF4 was not found to interact with HDA9 (van der Woude et al., 2019), PIF7 was recently identified as an interactor of HDA9 (Nguyen et al., 2023). Given PIF7’s critical role in TM and its capacity to heterodimerize with PIF4 (Kidokoro et al., 2009; Fiorucci et al., 2020), it’s possible that a PIF7/PIF4 heterodimer recruits the HDA9-PWR module to its target genes (Figure 1). The hypothesis of HDA9 recruitment to shade-induced genes via PIF7 has also been proposed for SAS (Nguyen et al., 2023).

During SM, HDA15 (HISTONE DEACETYLASE 15) is recruited by PIF3 to repress the expression of photosynthesis genes through H4 deacetylation (Liu et al., 2013). In addition, PIF1 also interacts with HDA15 to repress genes via histone deacetylation (H3ac) during seed germination in the dark (Figure 1) (Gu et al., 2017). In contrast to HDA9’s positive role, HDA15 is a negative TM regulator directly associating at thermo-responsive genes potentially through HFR1 (LONG HYPOCOTYL IN FAR-RED) (Shen et al., 2019). A current hypothesis that elucidates the contradictory functions of HDA9 and HDA15 proposes that at higher temperatures, the HFR1-HDA15 complex is displaced by PIF4, possibly forming a complex with HDA9 and PWR (Shen et al., 2019). HDA19 is an additional PIF1/3-interacting HDAC that represses PM via H3 deacetylation at the PM genes *BBX21* (*B-BOX CONTAINING PROTEIN 21*) and *GLK1* (*GOLDEN2-LIKE1*) (Guo et al., 2023). Another possible route of connecting PIFs with HDACs is HOS1 (HIGH EXPRESSION OF OSMOTICALLY RESPONSIVE GENE 1), a RING E3 ligase which can directly interact with PIF4 but also with HDA6 (HISTONE DEACETYLASE 6) and HDA15 (Jung et al., 2013; Kim et al., 2017).

An additional functional link between PIFs and HDACs are the subunits MED25 (MEDIATOR 25) and MED14 (MEDIATOR 14) of the Mediator complex (Bajracharya et al., 2022; Guo et al., 2023; Shapulatov et al., 2023), which is an evolutionary conserved large multi-subunit protein complex regulating RNAPII function on various levels (Allen and Taatjes, 2015; Soutourina, 2018). MED25 or PFT1 (PHYTOCHROME AND FLOWERING TIME 1) was first discovered as a SAS regulator and can interact with PIF4 in *Arabidopsis* and tomato to recruit RNAPII (Sun et al., 2020; Shapulatov et al., 2023). During TM, MED25 can associate with HDA9, thereby potentially facilitating the PIF4-mediated recruitment of HDA9 for histone deacetylation at the *PIF4* and *YUC8* gene (Figure 1) (Shapulatov et al., 2023). Moreover, PIF1 and PIF3 also interact with MED25 and HDA19 to down-regulate the expression of positive the positive PM regulators *BBX21* and *GLK1* via histone deacetylation and reducing chromatin accessibility (Figure 1) (Guo et al., 2023). Intriguingly, MED25 also undergoes LLPS to form biomolecular condensates with PIF1/3 and HDA19 (Guo et al., 2023). Moreover, PIF4 as well as its coactivator HMR (HEMERA) interact with MED14 to positively regulate thermo-responsive genes (Bajracharya et al., 2022).

Also noteworthy is the recently resolved protein composition of PBs, which has identified the Groucho/Tup1-type co-repressors TPL (TOPLESS) and TPR1 (TOPLESS-RELATED 1) as PB components (Kim et al., 2023). TPL and TPRs putatively exert their repressive function through the direct association with various HDACs such HDA6 and HDA19 (Long et al., 2006; Krogan et al., 2012; Wang et al., 2013; Plant et al., 2021). The confirmation of whether PIFs indeed interact with TPL/TPRs at their target genes remains uncertain at this point. However, the spatial proximity of PIFs and TPL/TPRs in PBs implies a potential functional connection (Kim et al., 2023). In addition, an interaction of PIF1 with LUH (LEUNIG_HOMOLOG), another member of the Groucho/Tup1-type co-repressor family, was shown to regulate expression of PIF1 target genes during seed germination through an unknown mechanism (Lee et al., 2015; Kim et al., 2023).

### Histone methylation dynamics

Methylation of various lysine (K) residues of histones H3 and H4 can occur in plants and depending on the modified lysine residue and degree of methylation (mono-, di-, and/or tri), gene expression is instructed differently (Liu et al., 2010; Xiao et al., 2016; Cheng et al., 2020). H3K4me3 on the +1 nucleosome is a critical epigenome feature indicating active genes, and assessing H3K4me3 occupancy is commonly employed as an indicator of an active chromatin state (Bernstein et al., 2002). Mutants in PIF-interacting CRs frequently exhibit altered levels of H3K4me3 at PIF target genes (Huai et al., 2018) However, whether this alteration is regulatory in nature or merely a consequence of transcriptional changes remains unclear. Until very recently, the exact function of H3K4me3 remained elusive. Using an elegant acute depletion method, all SET1/COMPASS complexes were eliminated from mouse embryonic stem cells, unveiling the critical involvement of H3K4me3 in regulating RNAPII pausing, elongation, and eviction (Wang et al., 2023).

The dark-to-light transition at the beginning of PM leads to a PIF-dependent rapid upregulation of gene expression and H3K4me3 levels (Calderon et al., 2022). How this achieved in a PIF-dependent manner is not clear but the PIF4 interacting transcriptional co-regulator SEU (SEUSS) might provide a link between PIFs and active H3K4me3 regulation (Figure 1) (Huai et al., 2018). SEU is a negative PM regulator under red, far-red, and blue light conditions, but interestingly a positive TM regulator (Huai et al., 2018). It has been demonstrated for SEUSS that it controls H3K4me3 deposition at *WOX5* (*WUSCHEL-RELATED HOMEOBOX 5*) targets genes by associating with the H3K4 methyltransferase SDG4 (SET DOMAIN GROUP 4) (Zhai et al., 2020). Therefore, it is plausible to hypothesize the existence of an operational PIF-SEU-SDG4 complex. Additionally, PIFs regulate the local H3K4me3 environment at their target genes by associating with INO80-C/COMPASS complexes during TM (Figure 1) (Shang et al., 2021; Xue et al., 2021).

Removal of histone methylation marks is facilitated by jumonji domain-containing histone demethylases (Lu et al., 2008; Mosammaparast and Shi, 2010). The primary demethylase in *Arabidopsis* is REF6 (RELATIVE OF EARLY FLOWERING 6) whose mutation causes ectopic gain of H3K27me3 at thousands of genes (Lu et al., 2011; Zander et al., 2019). REF6 was found to cooperatively regulate EAT-induced gene expression with PIF4 via H3K27me3 demethylation (Figure 1) (He et al., 2022). Whether REF6 can interact with PIFs is unknown but it can interact with INO80-C (Smaczniak et al., 2012), suggesting a potential regulatory pathway through which PIFs could influence the H3K27me3 landscape. During SM, PIF3 interacts with the SWI/SWF chromatin-remodeler (PKL/EPP1) (PICKLE/ENHANCED PHOTOMORPHOGENIC1) to repress H3K27me3 deposition at PIF3 target sites (Figure 1) (Zhang et al., 2014). How PKL inhibits the H3K27me3 deposition is not understood since PKL acts cooperatively with the SWR1-C ATPase PIE1 and the H3K27 methyltransferase CLF (CURLY LEAF) to establish and maintain the H3K27me3 landscape in *Arabidopsis* (Carter et al., 2018).

### Chromatin remodeling

Chromatin remodeling is a key process in genome organization, transcriptional regulation, DNA repair and replication (Clapier and Cairns, 2009). Of particular importance for gene expression is the accessibility of *cis*-regulatory elements and incorporation/eviction of histone variants which is regulated by multi-subunit ATP-dependent SWI2/SNF2 CRCs (Clapier and Cairns, 2009). Besides the interaction of PIFs with the INO80-type remodelers SWR1-C and INO80-C (Willige et al., 2021; Xue et al., 2021; Chen H. et al., 2023), PIFs also interact with various SWI/SNF-type remodelers (Zhang et al., 2017; Hussain et al., 2022). The SWI/SNF-type family consists of the BRM (BRAHMA)-, SYD (SPLAYED)-, and MINU1/2 (MINUSCULE1/2)-associated SWI/SNF (BAS, SASc (to avoid confusion with SAS), and MAS) complexes (Guo et al., 2022). PIF1 recruits BRM to photosynthesis genes in the dark to repress their expression though mechanism that remains unidentified (Zhang et al., 2017). Moreover, PIF7 interacts with AN3 (ANGUSTIFOLIA3), a subunit of either the BAS or SASc complex (Guo et al., 2022) to regulate leaf cell proliferation during shade (Hussain et al., 2022). Shade-stabilized PIF7 outcompetes AN3 at it target genes and represses their expression (Hussain et al., 2022). An additional antagonism between PIFs and a SWI/SNF subunit was shown for PIF4 and BAF60 (BRG1/BRM associated factor 60) (Jegu et al., 2017). BAF60 can be a subunit of the BAS, SASc, and MAS complex but because of a reported direct interaction between PIF4 and BRM, an association within the BAS complex is likely (Zhang et al., 2017; Guo et al., 2022). BAF60 target sites overlap with PIF4 DNA binding sites and strikingly BAF60 antagonizes PIF4 binding through the diurnal regulation of DNA accessibility at PIF4 binding sites (Figure 1) (Jegu et al., 2017).

## Discussion

Where in the gene, where in the nucleus, and where in the plant do the PIF-CR complexes act? For PIFs and their respective CRs to interact, they must be brought into proximity. Unlike CRs, which typically occupy gene bodies, PIFs span a wide spectrum, ranging from proximal to distal, intronic, and 3′ CRE binding (Chung et al., 2020; Willige et al., 2021). Most CRE-promoter communications are established by chromatin loops where TF-bound distal CREs or enhancers make direct physical with the proximal promoter/gene body region (Panigrahi and O'Malley, 2021). Indeed, a COP1 (CONSTITUTIVELY PHOTOMORPHOGENIC 1)-controlled phyB-PRC2 (POLYCOMB REPRESSIVE COMPLEX 2)-mediated chromatin loop has been identified at the positive SAS regulator *ATHB2* (Steindler et al., 1999; Kim et al., 2021; Wang et al., 2024), which is one of the most prominent shade and EAT-induced PIF7 targets, possessing an unusually large CRE with five PIF7 binding peaks 3–9 kb (kilobase) upstream of its transcription start site (Chung et al., 2020; Willige et al., 2021; Burko et al., 2022). Notably, the PIF interactor MED25 facilitates chromatin looping during active jasmonic acid (JA) signaling through interaction with the JA master TF MYC2 (Wang et al., 2019). Like MYC2 which can form tetramers to support loop formation (Lian et al., 2017), PIF4 can also form tetramers suggesting the potential of PIF4 to form loops (Gao et al., 2022).

This leads us to the nuclear 3D space and our second question: where precisely within the nucleus do the PIF-CR complexes act? We pose this question due to the rapid nature of shade-induced PIF7 DNA binding, which occurs at a few hundred genes within 5 min of shade exposure (Willige et al., 2021), potentially even earlier. Since we lack information regarding the DNA scanning speed of PIF7, we cannot ascertain whether the swift DNA targeting of PIF7 is attributable to PIF7’s search throughout the entire nucleus, or as previously speculated, solely in proximity to PBs (Van Buskirk et al., 2012). Various nuclear bodies in mammals are known to directly regulate chromatin activities (Shan et al., 2023), and intriguingly, a temperature-dependent chromatin association has been demonstrated for phyB (Jung et al., 2016). Moreover, recent findings have unveiled an active role of phyB in chromatin loop formation at the PIF target *ATHB2* (Kim et al., 2021; Wang et al., 2024). To further explore this exciting scenario of PB-associated chromatin structures, future research necessitates 3D chromatin conformation analyses as well as single locus imaging technologies.

All findings presented here stem from bulk-level analyses, which may obscure crucial spatial information (Cole et al., 2021). It has been demonstrated that for EAT-induced hypocotyl elongation, it is crucial for PIF4 to function within the epidermis (Kim et al., 2020), in conjunction with the DOF TF CDF2 (CYCLING DOF FACTOR 2) (Gao et al., 2022). Similarly, epidermal phyB plays a pivotal role in regulating most light responses, including SAS (Kim et al., 2016). Recent advancements in single-cell (sc) technologies, such as single-cell RNA sequencing (scRNA-seq), now facilitate the capturing of gene expression profiles at the single-cell level (Han et al., 2023). Thus far, only one scRNA-seq study has been reported for a *pif* mutant revealing that expression levels of *PIF1/3/4/5* remain relatively uniformly across cells in wildtype aerial tissues, but interestingly, the expression of PIF target genes varies among different cell types in *pifq* mutants (Han et al., 2023). This suggests that the cell-type specificity of PIF signaling may stem from cell type-specific epigenome disparities at PIF target genes (Han et al., 2023). Considering that PIFs initiate epigenome reprogramming at their target genes by recruiting various CR complexes, we hypothesize the existence of cell type-specific PIF-CR complexes to establish gene expression patterns unique to each cell type.

Highlighting these discoveries underscores the pivotal role of PIFs as epigenome landscapers. From our viewpoint, three key elements of PIFs’ epigenome landscaping abilities are particularly noteworthy. Firstly, binding of PIFs to CREs at their target genes is the starting point of stimulus-induced epigenome reprogramming. Secondly, most epigenome features can be directly and simultaneously governed by functionally diverse PIF-CR complexes. Thirdly, several PIF-CR modules, such as PIF-INO80-C, PIF-MRG1/2, and PIF-ASF1-HIRA are operational in multiple response pathways like SAS and TM (Shang et al., 2021; Willige et al., 2021; Yang et al., 2023; Zhao et al., 2023; Zhou et al., 2024), indicating that these complexes belong to the general repertoire of PIF-mediated transcriptional regulation.
